# Stereological estimation and morphological assessment of the endocrine pancreatic components in relation to sex in hen

**Published:** 2015-03-15

**Authors:** Rahmat Allah Fatahian Dehkordi, Hamid Moradi

**Affiliations:** 1 *Department of Anatomical Sciences, Faculty of Veterinary Medicine, University of Shahrekord, Shahrekord, Iran; *; 2 *DVM student, Faculty of Veterinary Medicine, University of Shahrekord, Shahrekord, Iran.*

**Keywords:** Native hen, Pancreas, Pancreatic islets, Sex, Stereology

## Abstract

In pancreatic survey, quantitative and morphological characteristics could be fundamental variables for the evaluation of some structures. The aim of this study was the application of stereological methods for estimation of quantitative parameters and morphological evaluation of the pancreas in chickens. Ten adult male and ten adult female native chickens were used in this study. Routine tissue processing was carried out for all samples. The sections were stained with hematoxylin and eosin and Gomori's aldehyde-fuchsin. Stereological examinations were performed according to systemic uniform random sampling as well as Cavalieri and point counting method. The morphological results showed that in both sexes, pancreas of chickens was composed of four main pancreatic lobes and contained two distinct islet types. Alpha islets consisted of alpha and a few delta cells and beta islets contained beta and seldom delta cells. Stereological results showed that in both sexes, there were significantly distinctive regional differences in the volume of islet and the numerical density of cells among some lobes with highest values in third and splenic lobes, respectively (*p *< 0.05). The nuclear volume was lowest in ventral lobe either males (80.60 ± 33.50) or females (84.20 ± 44.10). Beside, in the islets diameter of splenic lobe, there were significant differences in males as compared with females (*p *< 0.05). In splenic lobe, a significant difference was observed in the nuclear diameter in males than to females (*p *< 0.05). The results indicated that although there were not morphological changes between sexes in pancreatic lobes, however, some stereological parameters could be affected by sex.

## Introduction

The first studies shown that there are three pancreatic cell types (A, B and D cells) in most birds species.   ^1^^-^^7^ The avian pancreas can be divided into exocrine and endocrine parts, and the endocrine part can be further intersected into light and dark islet portions.   ^8^^-^^11^ Alpha islets stained with silver are known as dark islets, whereas beta islets not stained with silver are also known as light islets. ^12^^,^^13^ The secretions of exocrine part include many essential enzymes and many electrolytes. Whereas, the endocrine part is involved in control of blood sugar concentration.^   14 ^

In condition of the therapeutic, pathologic, aging and development, some quantitative estimates such as the volume of the islets and their volume-weight mean and the total cells number per islet, are the fundamental parameters in pancreatic structural studies. Beside, some authors have reported numerical and volumetric alterations of the pancreatic islets in diabetic subjects.   ^15^^-^^18^ 

Stereology is the first choice approach when three-dimensional data concerning structural quantities (volume, surface area, length and number) must be forecasted from tissue sections.   ^19^^-^^21^  Qualitative variables, including hyperplasia, hypoplasia and or hypertrophy, atrophy can be explained by stereological techniques through quantitative and comparable data.^   22 ^ Hence, the impact of different conditions on the alpha and beta cells survival or their changes needs simple and quick procedures of quantification. To understand the performance of islet cellular population, particularly during changes in islet cell mass, it is important to have dependable estimators among parameters that describe the quantitative appearance of the islet population.^4^^,^^16^^,^^18^ 

The aim of the present study was to investigate whether quantitative parameters of the endocrine pancreas, for instance, the islet volume and diameter, the total cells number of islets, show significant alterations between both sexes in chicken with the clear design of basic information concerning the stereological background of pancreatic islets.

## Materials and Methods

**Animals** **and** **tissue** **preparation.** Ten male (weighing 1840 ± 26 g) and ten female (weighing 2020 ± 45 g) adult healthy native hen were used in this study. Briefly, birds were maintained under similar conditions and kept in collective cages. Then, received drinking and bird’s food throughout the period (they received food and drink *ad libitum*). After one week, birds were sacrificed by an overdose (200 mg kg^-1^) intraperitoneal injection of sodium pentobarbital (Sigma, St. Louis, USA).

Abdominal cavity was exposed and after topographic examinations, entire pancreatic lobes were removed. For morphological studies, the pancreas was immediately fixed in Bouin’s solution (Dako North America, Carpinteria, USA) for 24 hr. Samples were processed by routine and standard paraffin embedding and serially sectioned in 5μm thickness. Tissue sections were deparaffinized and stained with Hematoxylin and Eosin and Gomori's aldehyde-fuchsin. 

**Fig. 1:**
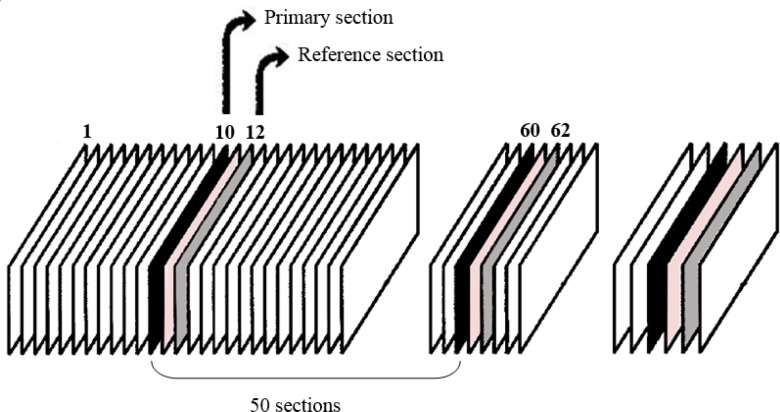
The sampling of histological sections shown in this figure, the pancreas thoroughly was sectioned and sections of the primary and reference were used in stereological estimation.

**Stereological analysis. **Stereological studies on the serial sections were carried out by systemic uniform random method. ^16^^,^^23^ Before it, the primary volume of the pancreas was calculated according to Cavellieri’s method.^   24 ^ According to random number's table, in the distance between the 50 sections the first section, which is known as the primary section (e.g., 10^th^ section, Fig. 1), and the second section that called reference section were selected (e.g., 12^th^ section, Fig. 1). Sampling interval between sections was so that each 50^th^ section was sampled (the primary sections). Therefore, for the primary and reference sections, e.g. 10, 12, 60, 62 were sampled in the entire pancreas. ^16^^,^^23^ Although for cell recognition, immunostain was applied to visualize insulin, but aldehyde fuchsin was used for all primary and reference sections here.

Sections from the pancreas were systematically analyzed by point-counting grid. Each islet was characterized by normal islet cells that considered as a defined islet for analysis. The number of test points (intersections on grid) overlying the tissue component of interest was counted. According to Cavellieri’s method, the total volume of islets was calculated. The total volume of islets was calculated using the following formula:^   23 ^

$V\left(\mathrm{isl}\right)=a/p(\mathrm{isl})\times N_{\mathrm{sect}}(p-p)\times T_{\mathrm{sect}}\times \Sigma P(\mathrm{isl})$

where, V(*isl*) is the all volume of islets, a/p(*isl*) is the area per point that *Δx × Δy*/*100* and *ƩP(isl)* is the all number of points that hit the islets. 

The numerical density of cells, *N(AC)*, were calculated by the following formula:

N(AC)=N(isl)/A(isl)

where, *N(isl)* is the number of nuclei per islet, and *A(isl)* is islet area.^   25 ^

Axes of the islets were measured by X (*a*) and Y (*b*) axis using a cross-calibrated micrometer, The diameter of the islets (*d*_i_) was calculated from the following equation.^   26 ^

$d_{i}=\sqrt[{2}]{\mathrm{ab}}$

To calculate the mean nuclear volume were used from the mean nuclear diameter (*Dn*).^26^

$V=4\pi /3\times {\left(\overset{\text{̅}}{D}/2\right)}^{3}$

where, *D* is nuclear diameter. 

**Statistical analysis. **All findings were analyzed using SPSS (Version 11.5; SPSS Inc., Chicago, USA). Stereological parameters were compared by one-way ANOVA, and Tukey’s test was used as a post-hoc test. Differences were considered as significant when *p* values were less than 0.05.

## Results

**Fig. 2:**
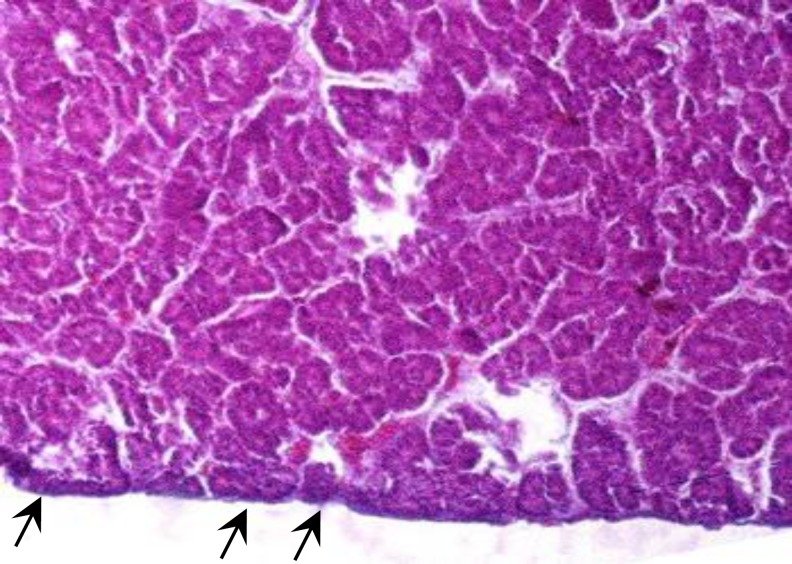
Photomicrograph of pancreas in male chicken. Note to pancreas capsule consisted of dense connective tissue (arrows), (H & E, 20×).

**Fig. 3:**
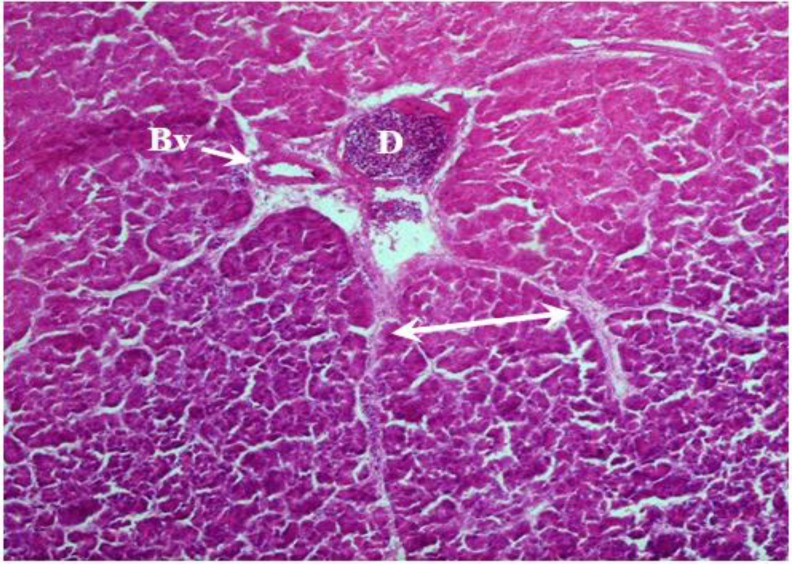
Photomicrograph of pancreas in male chicken. Intralobular duct (**D**) and blood vessel (**Bv**) observed between pancreatic lobules with connecting tissue septa (bilateral arrow), (H & E, 20×).

**Fig. 4:**
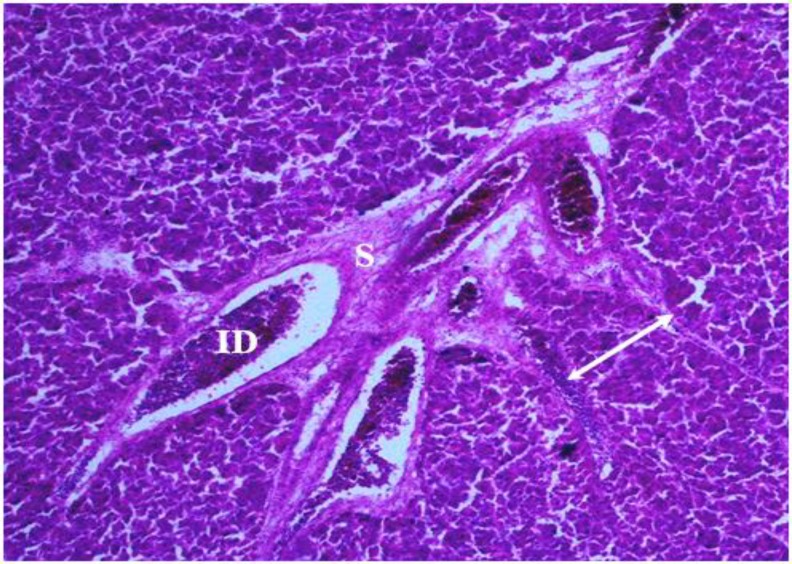
Photomicrograph of pancreas of male chicken. Note the intrelobular duct (**ID**) and pancreatic septum (**S**) that has been situated between pancreatic lobules (bilateral arrow), (Gomori’s aldehyde-fuchsin, 20×).

**Morphological results. **In the present study, the pancreas capsule was composed of dense connective tissue that surrounded around the organ and penetrated into the gland (Figs. 2, 3 and 4). In both sexes, the chickens pancreas of interest were found to be located between the duodenal loops and can be divided into dorsal, ventral, third and splenic lobes. Third lobe was a branch that originated from ventral lobe and included elongation axis of the longitudinal.

**Fig. 5:**
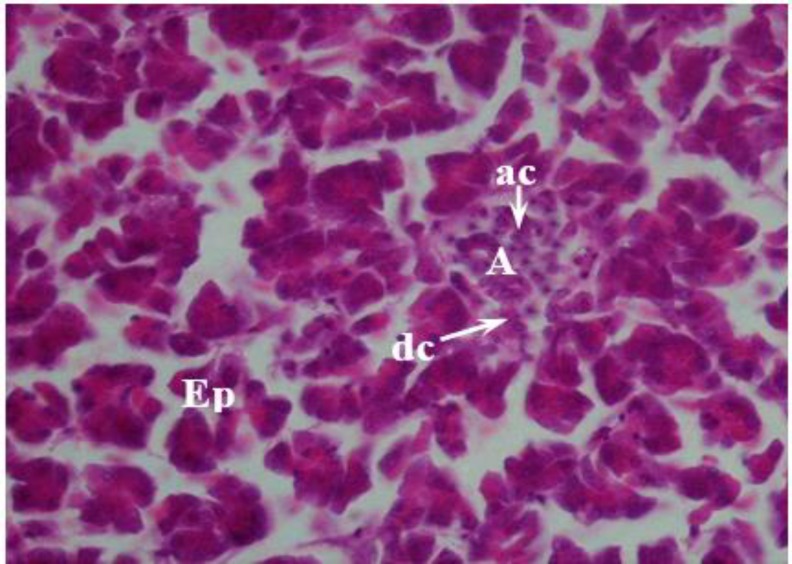
Photomicrograph of pancreas in female chicken. Endocrine pancreas, alpha islet (**A**) into exocrine pancreas (**Ep**), alpha cell (**ac**), delta cell (**dc**), (H & E, 40×).

**Fig. 6:**
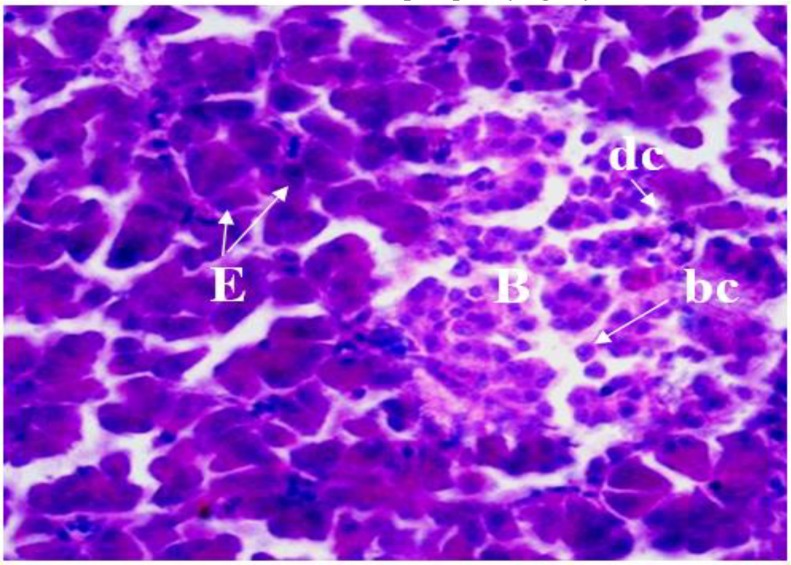
Photomicrograph of pancreas in female chicken. Note the endocrine pancreas, beta islet (**B**) and acinar cells of pancreas (**E**), beta cell (**bc**), delta cell (**dc**), (Gomori’s aldehyde-fuchsin, 40×).

In all the lobes, two type islets (alpha and beta islets) were clearly distinguishable from each other (Figs. 5 and 6). Alpha islets were thoroughly distributed in all the glandular lobes, as those were similar in beta islets, which exist in the principal lobes. The islets were also variable and moderate in the size and shape in the alpha and beta respectively (Figs. 5 and 6). In the alpha islets, the alpha cells had a columnar shape, round to oval in shape nucleus containing one or two distinguished nucleolus (Fig. 5). 

Despite alpha cells, which were functionally distinct populations, alpha islets were surrounded by small cells, which were oval and ring-shaped as named delta cells. 

In the beta islets, the beta cells with a polygon or elliptical shape, with a spherical nucleus that was enclosed with the slight and scattered chromatin. The beta cells were stained by Gomori's aldehyde-fuchsin, and they showed a dark bluish color to purple (Fig. 6).

**Stereological results. **Stereological characteristics in different parts of the chicken pancreas are summarized in Table 1. For the total studied parameters, statistical analysis displayed different variations between male and female chickens (*p* < 0.05) for each parameter alone. 

**Table 1:** The mean volume of islets, islet diameter, number of cells per islet, and nuclear volume and diameter in chicken. Values are presented as means ± SEM

**Parameters**	**Sex**	**Dorsal lobe**	**Ventral lobe**	**Third lobe**	**Splenic lobe**
**Volume of islets (** ***µm*** ^3^ **)**	Male	2.50 ± 0.11	2.25 ± 0.10	2.80 ± 0.11*	2.40 ± 0.12
	Female	2.48 ± 0.10	2.30 ± 0.09	2.70 ± 0.11*	2.50 ± 0.10
**Islets diameter (** ***µm*** **)**	Male	69.10 ± 4.30	64.30 ± 3.60	79.40 ± 5.50	89.60 ± 3.20**
	Female	67.30 ± 3.70	63.80 ± 2.79	79.08 ± 4.80	80.40 ± 3.10**
**Numerical density of cells**	Male	296.00 ± 30.21	342.70 ± 37.40	284.22 ± 28.90	453.2 ± 42.30*
	Female	289.31 ± 30.40	322.90 ± 34.20	276.50 ± 27.30	431.60 ± 40.37*
**Nuclear volume** **(*µm***^3^**)**	Male	98.35 ± 54.30	80.60 ± 33.50	101.20 ± 58.40	97.36 ± 45.60
	Female	86.90 ± 45.20	84.20 ± 44.10	104.20 ± 50.01	99.40 ± 32.00
**Nuclear diameter (** ***µm*** **)**	Male	5.30 ± 0.13	6.10 ± 0.21	6.30 ± 0.14	6.20 ± 0.13**
Female	5.31 ± 0.12	5.80 ± 0.11	6.30 ± 0.10	5.41 ± 0.11**

* indicates significant difference compared to the other lobes in the same row (*p* < 0.05).

** indicates significant difference in male compared to female in the same parameter (*p* < 0.05).

In both sexes, the volume of islets were increased from the minimum of 2.25 ± 0.10 (male) and 2.30 ± 0.09 (female) in ventral lobe to the maximum of 2.80 ± 0.11 (male) and 2.70 ± 0.11 (female) in third lobe. The statistical survey showed that there was significant difference in the islets volume of third lobe than to three other lobes (*p* < 0.05). In all the lobes, there was no significant difference in the islets volume between male compared to female (*p *> 0.05).

The mean of the islets diameter did not show noticeable difference among four pancreatic lobes in both sexes, except for splenic lobe that showed a large increase in male chickens (89.60 ± 3.20, male; 80.40 ± 3.10, female). However, a significant difference was seen in islets diameter of splenic lobe in males compared to females (*p *< 0.05).

These results showed that the maximum numerical density of cells occurred in splenic lobe, 453.20 ± 42.30 male and 431.60 ± 40.37 in female, and the minimum was observed in third lobe, 284.22 ± 28.90 male and 276.50 ± 27.30 in female. Besides, there was a significant difference in numerical density of cells of splenic lobe compared to other lobes (*p *< 0.05).

In both sexes, there was no significant difference among four pancreatic lobes in value of the nuclear volume. Also in parameter of interest, significant changes were not seen between males than females in all pancreatic lobes (*p *> 0.05).

The mean of nuclear diameter of islets did not show significant difference among all lobes in both sexes (*p *> 0.05), however, a significant change was observed in the values of nuclear diameter of splenic lobe in males compared to females (*p *< 0.05).

## Discussion

The present study showed a similar morphology pattern in pancreatic gland of the male and female native chickens. To our knowledge, no sex differences in morphologic properties of the pancreas gland have been reported in birds. 

The results from the morphologic findings showed that in both sexes of native chickens, in addition to dorsal, ventral and third lobes in the pancreas, there was a splenic lobe in both sexes. This finding was in agreement with reports of the other investigators in some birds. Similar results was found in goose^9^ and duck^       27 ^ but was reversed about‎ lobes of the mynah’s pancreas.^3^ According to observations of the Saadatfar and Asadian, it was detected which mynah’s pancreas had no splenic lobe.^3^ In goose, the pancreas was located between the duodenal loops and also there were dorsal, ventral, third and splenic lobes.^9^ Mikami and Ono showed that there were individual ducts in the dorsal, ventral and third lobes,^   13 ^ an also it was observed that the third lobe occasionally consisted of its own duct.^   28 ^

The present study showed that the third lobe, with the elongation axis along the longitudinal, was a branch that was originated from ventral lobe as described by Saadatfar and Asadian in mynah’s pancreas.^3^ In the relationship with the tissue sections of the chicken pancreatic islets, it was revealed that there are two types of islets, alpha and beta that contain three types of general cells, alpha, beta and delta. Alpha islets did not indicat borders with the exocrine parts, but beta islets were distinctly separated from the exocrine part by connective tissue. In white Leghorn chickens, Oakberg showed that usually alpha and beta cells lie in disconnected and individual islets. They showed that in the Leghorn chickens, when both alpha and beta cells were put together in one islet, beta cells form compact groups were encircled by alpha cells.^   29 ^ Alpha cells into alpha islet were in peripheral regions as some researchers reported these cells occupy circumference of the islets.^9^ In an immunocytochemical study, Cowap displayed that somatostatin cells were found in all splenic lobes of pancreas.^5^

Stereological findings of this study indicated basic quantitative characteristics of the chicken pancreas and their changes in different lobes of both sexes. The present study showed that there were some variations in the quantitative parameters such as the volume of islets, the islets diameter, the numerical density of cells, nuclear volume and diameter, which may be related to different degrees of islets activity in the pancreas. In addition, there were regional differences in quantitative values of interest in both sexes. Elayat *et al*. showed that distinct regional differences existed in the rat pancreatic islets. They showed that these alterations existed not only among the ventral and dorsal lobes, but also between the regions of gastric and splenic lobes.^   25 ^ It seems that existence of different pancreatic primordia, during development, causes regional differences in the pancreatic islets.^30^^,^^31^ 

The stereological data showed that the maximum quantitative variable rate was associated with the spleen lobe, but the reverse was true for other pancreatic lobes. In agreement with our results, some previous studies showed special role of the splenic lobe in examinations of the pancreas. ^10^^,^^32^  There was significance in the islets diameter, the numerical density of cells and the nuclear diameter between males compared to females. Herbach *et al*. showed that there was no significant difference concerning the development of the total islet and islets cells volume between male and female animals.^   33 ^ In some of the pathological conditions, such as diabetes mellitus and islet cell tumors, quantitative data provides proper perspective for better understanding of such diseases as well.   ^16^^,^^25^^,^^34^ 

In conclusion, morphological study of the pancreas islets revealed that the structure of native chicken pancreas was similar to that of other avian species. Furthermore, our quantitative data showed that basic stereological characteristics of endocrine pancreas in chicken and indicated that the structure of chicken pancreas was partially affected by sex in some lobes.

## References

[1] Jain DK, Goel R (1994). Immunocytochemical localization of endocrine, paracrine and neurocrine cells in the avian pancreas. J Appl Anim Res.

[2] Mobini B (2009). A Preliminary histomorphometrical study on pancreas of duck. J Appl Anim Res.

[3] Saadatfar Z, Asadian M (2009). Anatomy of pancreas in mynah (Acridotheres tristis). J Appl Anim Res.

[4] Bonner-Weir S, Weir G (1979). The organization of the endocrine pancreas: A hypothetical unifying view of the phylogenetic differences. Gene Comp Endocrinol.

[5] Cowap J (1985). The first appearance of endocrine cells in the splenic lobe of the embryonic chick pancreas. Gene Comp Endocrinol.

[6] Larsson LI, Sundler F, Hakanson R (1974). Localization of APP, a postulated new hormone, to a pancreatic endocrine cell type. Histochem Cell Biol.

[7] Rawdon B, Andrew A (1979). An immunocytochemical study of the distribution of pancreatic endocrine cells in chicks, with special reference to the relationship between pancreatic polypeptide and somatostatin-immuno-reactive cells. Histochem Cell Biol.

[8] Gulmez N (2003). Are glands present in goose pancreatic ducts? A light microscope study. J Pancrease.

[9] Gulmez N, Kocamis H, Aslan S (2004). Immuno-histochemical distribution of cells containing insulin, glucagon and somatostatin in the goose (Anser anser) pancreas. Turk J Vet Anim Sci.

[10] Smith PH (1974). Pancreatic islets of the Coturnix quail A light and electron microscopic study with special reference to the islet organ of the splenic lobe. Anat Rec.

[11] Buchan AM, Lance V, Polak JM (1982). The endocrine pancreas of Alligator mississippiensis. Cell Tissue Res.

[12] Hodges RD (1974). The Histology of the fowl.

[13] Mikami SI, Ono K (1962). Glucagon deficiency induced by extirpation of alpha islets of the fowl pancreas. Endocrinology.

[14] Aughey E, Frye FL (2001). Comparative veterinary Histology with clinical correlates.

[15] Paulsen SJ, Vrang N, Larsen LK (2010). Stereological assessment of pancreatic beta‐cell mass development in male Zucker diabetic fatty (ZDF) rats: correlation with pancreatic beta-cell function. J Anat.

[16] Bock T, Pakkenberg B, Buschard K (2003). Increased islet volume but unchanged islet number in ob/ob mice. Diabetes.

[17] Mahmoudzadeh-Sagheb H, Heidari Z, Shahraki M (2010). A stereological study of effects of aqueous extract of Tamarindus indica seeds on pancreatic islets in streptozotocin-induced diabetic rats. Pak J Pharm Sci.

[18] Skau M, Pakkenberg B, Buschard K (2001). Linear correlation between the total islet mass and the volume-weighted mean islet volume. Diabetes.

[19] Mayhew T (1992). A review of recent advances in stereology for quantifying neural structure. J Neurocytol.

[20] Mayhew T (1991). The new stereological methods for interpreting functional morphology from slices of cells and organs. Exp Physiol.

[21] Cruz-Orive LM, Weibel ER (1990). Recent stereological methods for cell biology: a brief survey. Am J Physiol-Lung Cell Mol Physiol.

[22] Noorafshan A, Hoseini L, Karbalay-Doust S (2012). A Simple stereological method for estimating the number and the volume of the pancreatic beta cells. JOP J Pancreas.

[23] Bock T, Pakkenberg B, Buschard K (2005). Genetic background determines the size and structure of the endocrine pancreas. Diabetes.

[24] Howard C, Reed M (1998). Estimation of reference volume using the Cavalieri method Unbiased Stereology Three-dimensional measurement in microscopy.

[25] Elayat AA, El-Naggar MM, Tahir M (1995). An immunocytochemical and morphometric study of the rat pancreatic islets. J Anat.

[26] Williams MA (1977). Quantitative methods in biology: Practical methods in electron microscopy.

[27] McClish RD, Eglitis JA (1969). Distribution of the A and B Cells and of the islets (Langerhans) in the duck pancreas. The Ohio J Sci‎.

[28] McLeod WM, Trotter D, Lumb J (1964). Avian anatomy.

[29] Oakberg EF (2005). Quantitative studies of pancreas and islands of Langerhans in relation to age, sex, and body weight in white Leghorn chickens. Am J Anat.

[30] Guz Y, Montminy M, Stein R (1995). Expression of murine STF-1, a putative insulin gene transcription factor, in beta cells of pancreas, duodenal epithelium and pancreatic exocrine and endocrine progenitors during ontogeny. Development.

[31] Baetens D, Malaisse-Lagae F, Perrelet A (1979). Endocrine pancreas: Three-dimensional reconstruction shows two types of islets of Langerhans. Science.

[32] Simsek N, Alabay B (2008). Light and electron microscopic examinations of the pancreas in quails (Coturnix coturnix japonica). Rev Med Vet.

[33] Herbach N, Goeke B, Schneider M (2005). Overexpression of a dominant negative GIP receptor in transgenic mice results in disturbed postnatal pancreatic islet and beta-cell development. Regul peptides.

[34] Herbach N, Bergmayr M, Göke B (2011). Postnatal development of numbers and mean sizes of pancreatic islets and beta-cells in healthy mice and giprdn trans-genic diabetic mice. PLoS One.

